# Updating “machine learning imagery dataset for maize crop: A case of Tanzania” with expanded data to cover the new farming season

**DOI:** 10.1016/j.dib.2024.110359

**Published:** 2024-03-23

**Authors:** Neema Mduma, Flavia Mayo

**Affiliations:** Nelson Mandela African Institution of Science and Technology, Box 447 Tengeru, Arusha, Tanzania

**Keywords:** Machine learning, Smart agriculture, Disease monitoring, Farmers, Tanzania

## Abstract

Maize Lethal Necrosis (MLN) and Maize Streak Virus (MSV) are among maize diseases which affect productivity in Tanzania and Africa at large. These diseases can be detected early for timely interventions and minimal losses. Machine learning (ML) has emerged as a powerful tool for automated diseases detection, offering several advantages over traditional methods. This article presents the updated dataset of 9356 imagery maize leaves to assist researchers in developing technological solutions for addressing crop diseases. The high-resolution imagery data presented in this dataset were captured using smartphone cameras in farm fields which were not selected in the previously published dataset. Also, data collection was taken in the range of three months from November 2022 to January 2023 to incorporate farming season not covered in the previously published dataset. The presented dataset can be used by researchers in the field of Artificial Intelligence (AI) to develop ML solutions and eliminate the need of manual inspection and reduce human bias. Developing ML solutions require large amount of data therefore, the updated and previously published datasets can be combined to accommodate diverse and wider applicability.

Specifications Table

**Table:** 

Subject	Artificial Intelligence.
Specific subject area	Detecting crop diseases.
Data format	Raw
Type of data	Image
Data collection	Same as in original data article
Data source location	Tanzania Agricultural Research Institute (TARI) and Nelson Mandela African Institution of Science and Technology (NM-AIST), Tanzania
Data accessibility	Mendeley 10.17632/fkw49mz3xs.1 https://data.mendeley.com/datasets/fkw49mz3xs/1
Related data article	N. Mduma, H. Laizer, Machine Learning Imagery Dataset for Maize Crop: A Case of Tanzania, Data in Brief 48 (2023) 1–4. https://doi.org/10.1016/j.dib.2023.109108

## 1 Value of the Data

• The updated dataset introduces maize leaf images captured in different farming season, unlike the prior dataset.
• Researchers can use the presented dataset to develop disease monitoring systems that track the spread of MSV and MLN in real-time, allowing for targeted interventions and prevention strategies.
• The updated dataset includes maize leaf images captured using a high-resolution smartphone camera, making the technology accessible to a wider range of farmers.

## 2 Data Description

Agricultural sector forms the core of Tanzania's economic activity, employing over four out of five citizens and serving as their essential means of life [1]. Nearly one-third of Tanzania's economic output, as measured by GDP, originates from agriculture, which also employs over two-thirds of the workforce [2]. Maize is among the dominant food and cash crops in the country, accounting for about 45 % of its farmland [3]. Despite its importance to Tanzania's economy and food security, maize is hindered by diseases, specifically Maize Lethal Necrosis (MLN) and Maize Streak Virus (MSV), which highly affect its productivity [4,5]. Therefore, effective crop disease control strategies are essential for optimal productivity.

Several management methods have been implemented for these diseases, including chemical treatment and human decisions based on physical observations. However, these methods are based on human judgments and considered unreliable with bias. On the other hand, deep learning tools have been developed to help stakeholders in agriculture and food systems to detect crop diseases early [6,7]. This approach can assist in the early detection of maize diseases for proper management. However, developing deep learning tools requires enormous data, and Tanzania lacks an original dataset to facilitate model development. To address this issue, the dataset of maize leaf images was published in the Harvard repository in March 2023 [8]. The dataset consisted of 18,148 images (Healthy = 5118; MSV = 6255; MLN = 3982) collected from February to July 2021. The dataset was reported to contains all possible instances however, it did not capture the data samples collected in all farming seasons. Therefore, this article presents an updated dataset of maize leaf images incorporating the missing farming season data.

**Fig. 1:**
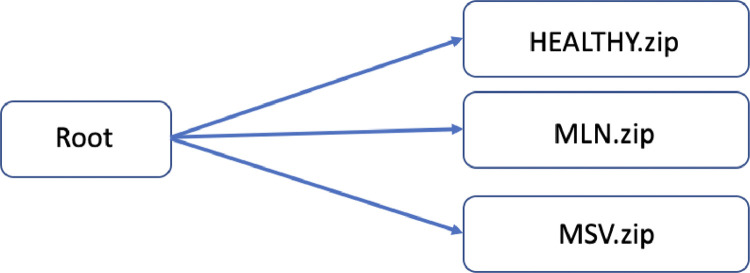
Structure of the data repository.

The updated dataset of 9356 images was divided into three classes (Healthy = 3073 images; MSV = 3052 images; MLN = 3231 images) and published in an open-access repository on February 22, 2023 [9]. The repository contains three separate zipped folders (HEALTHY.zip, MLN.zip, and MSV.zip) to allow users to download a specific file or all files simultaneously based on their preferences. HEALTHY.zip folder has 2.34 GB, MLN.zip folder has 2.23 GB and MSV.zip has 2.4 GB. A visual representation of the data repository's structure is presented in Fig. 1.

## 3 Experimental Design, Materials and Methods

### 3.1 Data collection

**Fig. 2:**
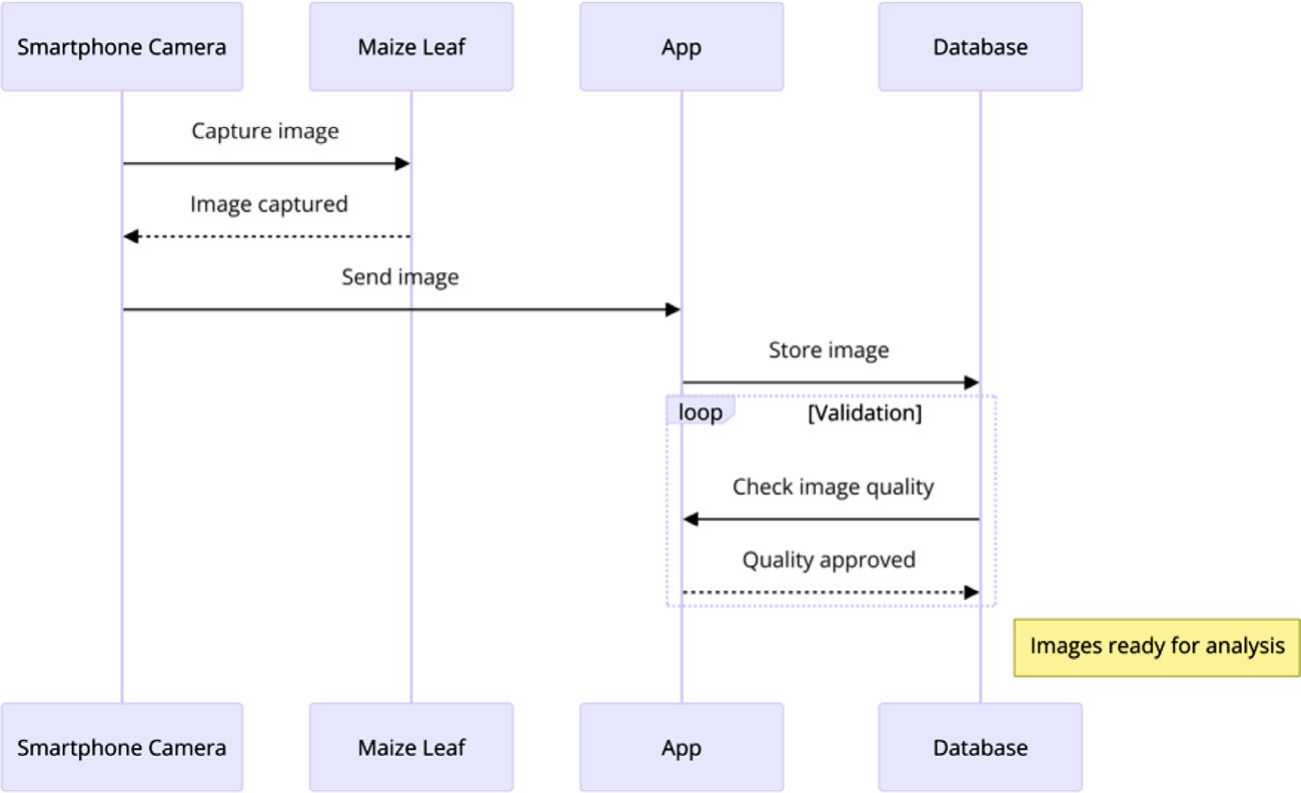
Sequence diagram of the data collection process.

Data collection tool was installed on a smartphone (iPhone XS Max) and used to collect maize imagery leaves. The images were sent to the database for the quality check before data analysis as seen in Fig. 2. The data collection process involved farmers and experts from the Northern Tanzania, particularly the Tanzania Agricultural Research Institute (TARI) and Nelson Mandela African Institution of Science and Technology (NM-AIST) in Arusha. Maize imagery data were collected from TARI farms in Arusha from November 2022 to January 2023. Site selection for data collection prioritized areas with readily available maize crops and demonstrable occurrences of MLN and MSV disease symptoms. The farms with diseased plants were identified with the help of plant pathologists and agricultural experts.

### 3.2 Data preprocessing

The collected images were preprocessed, and this included data cleaning and labelling. VisiPics tool removed duplicate images from all three classes (Healthy, MSV, and MLN) [10]. The images were also labelled to correspond to the class name, i.e., HEALTHY_876.jpg, using the bulk rename utility. The dataset consists of images in Joint Photographic Experts Group (JPG) format in different sizes.

## Limitations

The dataset presented in this article contains maize imagery leaves specifically collected in Arusha. The dataset is, however, limited to only two diseases: MLN and MSV. The main focus was to ensure the dataset contains high-quality imagery data which would otherwise lead to bias; therefore, the dataset is limited to images acquired under well-lit conditions and excludes those captured with low-quality smartphone cameras. Future datasets can explore the possibility of including other regions in Tanzania not covered during the data collection and incorporating other maize diseases affecting productivity.

## Ethics Statement

Data for this study was gathered from farms with informed consent obtained through a formal approval process. Farmers actively participated by completing consent forms, granting permission for data collection on their land.

## CRediT authorship contribution statement

**Neema Mduma:** Investigation, Formal analysis, Resources, Validation, Writing – original draft, Writing – review & editing, Visualization, Software, Supervision, Project administration, Methodology, Funding acquisition, Conceptualization. **Flavia Mayo:** Conceptualization, Writing – review & editing, Methodology, Data curation.

## Data Availability

Maize Imagery Dataset - Tanzania (Original data) (Mendeley Data).
